# Effect of granulocyte colony stimulating factor (G-CSF) on IVF outcomes in infertile women: An RCT 

**Published:** 2016-05

**Authors:** Maryam Eftekhar, Robabe Hosseinisadat, Ramesh Baradaran, Elham Naghshineh

**Affiliations:** 1 *Research and Clinical Center for Infertility, Shahid Sadoughi University of Medical Sciences, Yazd, Iran.*; 2 *Department of Obstetrics and Gynecology, School of Medicine, Kerman University of Medical Sciences, Kerman, Iran. *; 3 *Department of Obstetrics and Gynecology, School of Medicine, Isfahan University of Medical Sciences, Isfahan, Iran.*

**Keywords:** *G-CSF*, *In vitro fertilization*, *Pregnancy rates*, *Randomized controlled trial*

## Abstract

**Background:**

Despite major advances in assisted reproductive techniques, the implantation rates remain relatively low. Some studies have demonstrated that intrauterine infusion of granulocyte colony stimulating factor (G-CSF) improves implantation in infertile women.

**Objective:**

To assess the G-CSF effects on IVF outcomes in women with normal endometrial thickness.

**Materials and methods:**

In this randomized controlled clinical trial, 100 infertile women with normal endometrial thickness who were candidate for IVF were evaluated in two groups. Exclusion criteria were positive history of repeated implantation failure (RIF), endocrine disorders, severe endometriosis, congenital or acquired uterine anomaly and contraindication for G-CSF (renal disease, sickle cell disease, or malignancy). In G-CSF group (n=50), 300 µg trans cervical intrauterine of G-CSF was administered at the oocyte retrieval day. Controls (n=50) were treated with standard protocol. Chemical, clinical and ongoing pregnancy rates, implantation rate, and miscarriage rate were compared between groups.

**Results:**

Number of total and mature oocytes (MII), two pronuclei (2PN), total embryos, transferred embryos, quality of transferred embryos, and fertilization rate did not differ significantly between two groups. So there were no significant differences between groups in chemical, clinical and ongoing pregnancy rate, implantation rate, and miscarriage rate

**Conclusion:**

our result showed in normal IVF patients with normal endometrial thickness, the intrauterine infusion of G-CSF did not improve pregnancy outcomes.

## Introduction

Despite major progression in assisted reproductive techniques, the implantation rates still remain relatively low. “Successful implantation needs good quality embryo, receptive endometrium, and good embryo transfer technique” (1). The receptive endometrium is a healthy uterine milieu that support the transformation of endometrial cells into decidua cells, invasion of blastocysts, and rapid growth of placenta (2). This process is facilitated by immune cells, growth factors, cytokines, and hormonal changes (3, 4).

Immunological mechanisms in the endometrium are very important and crucial in implantation process (5). Granulocyte colony-stimulating factor (G-CSF) is a hematopoietic cytokine produced in maternofetal interface during embryo implantation and early pregnancy suggesting it may play a role in decidua and placental function (6). It stimulates granulocyte proliferation and differentiation (7). 

Some studies have demonstrated that systemic administration of G-CSF in women with recurrent spontaneous abortions and repetitive implantation failures improves pregnancy outcomes (8-10). Also, G-CSF transvaginal infusion successfully were used in women with thin endometrial thickness (<7 mm) and repetitive implantation failures recently (11, 12). It should be duo to improving endometrial thickness after G-CSF administration (13). Eftekhar *et al* showed intrauterine G-CSF administration improved chemical and clinical pregnancy rate in infertile women with thin endometrium in frozen-thawed embryo transfer cycles but they found in their study endometrial thickness in their patients did not increased (6). Fewer studies have examined the G-CSF effect in women with normal endometrial thickness. Barad *et al* demonstrated that intrauterine G-CSF infusion in fresh embryo transfer cycles in women underwent IVF treatment did not affect on endometrial thickness, implantation, and clinical pregnancy rates (7). Therefore, it is hypothesized that G-CSF inflammatory and immunological effects may improve the implantation rate and endometrial receptivity in infertile women 

In this study, G-CSF effect on implantation and pregnancy rates in normal infertile women were investigated. 

## Materials and methods

This randomized clinical trial was performed between March and September 2015 in Yazd Research and Clinical Center for Infertility. Study protocol was approved by Ethics Committee of Research and Clinical Center for Infertility, Yazd, Iran. 

100 infertile women aged 18-40 years old with normal endometrial thickness who were candidate for IVF were participated in this study (n=50 each group). Women with repeated implantation failure (RIF) (failure to conceive following two embryo transfer cycles, or cumulative transfer of >10 good-quality embryos), endocrine disorders, severe endometriosis, congenital or acquired uterine anomaly (uterine polyp, sub mucosal myoma, intrauterine adhesions), contraindication for G-CSF (renal disease, sickle cell disease, or malignancy history, upper respiratory tract infection, pneumonia, or chronic neutropenia) were excluded. 

After receiving informed written consent from all participants and their spouse, according to enveloped pocket method women were allocated randomly in two groups (G-CSF and control group). Standard agonist or antagonist protocol was used for ovarian stimulation in groups (14). When at least two follicles achieved 17 mm diameter, Human chorionic gonadotropin (hCG) (Choriomon 10000 IU, IBSA Institute, Switzerland) was administered for final oocyte maturation. Transvaginal oocyte retrieval was performed 36 hr after hCG injection. The oocytes were fertilized by intracytoplasmic sperm injection method. 

In G-CSF group at the day of oocyte retrieval, after oocytes collection, 300 mg G-CSF (300 µg/mL, Zahravi Co, Tehran, Iran) was administered by slow transcervical intrauterine infusion with IUI catheter (AINSEGREY, RIMOS, Italy) (6). In controls, the cycle were continued without G-CSF infusion. In all patients, 2-3 embryos were transferred by using embryo transfer catheter (Cook USA), two days after oocyte retrieval.

Pregnancy outcomes were assessed based on positive serum βhCG test (chemical pregnancy), 14 days after embryo transfer and observation of gestational sac on transvaginal ultrasound examination (clinical pregnancy), three weeks after positive serum βhCG. Implantation rate was assessed by the number of gestational sacs divided by the number of transferred embryos in each group. The ongoing pregnancy rate was defined as the presence of fetal heart activity by ultrasonography after 12 wks of pregnancy. The miscarriage rate was assessed by the number of miscarriages before 20 wks gestation per number of women with positive βhCG test.

**Statistical analysis**

Data were analyzed using Statistical Package for the Social Sciences 20.0 (SPSS, SPSS Inc, Chicago, Illinois). With 95% confidence level, power of 80%, p_1_=20%, p_2_=45% and the sample size=50 in each group was considered. Continuous data were presented as mean±SD and assessed by independent Student’s *t*-test. Qualitative data were compared by  ^2^ or fisher exact test. P<0.05 was considered significant.

## Results

**Figure 1:**
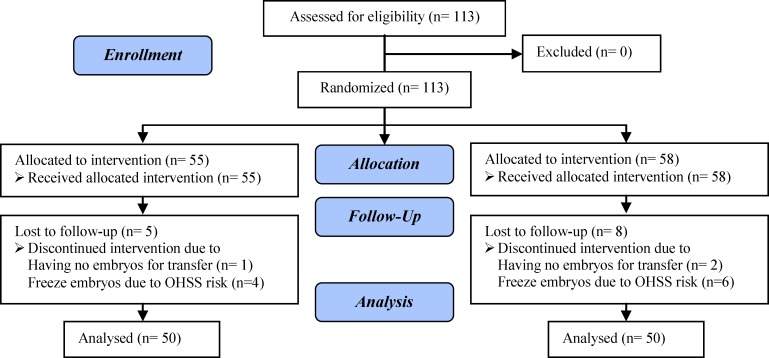
Consort flow diagram

Totally, 113 normal infertile women were participated in this study. 13 women were excluded and finally data of 100 women analyzed (Figure 1). Demographic characteristics of participants are presented in Table I. Two study groups matched for age, etiology, duration, and infertility type, number of previous embryo transfer cycles, and basal FSH level. There were no significant differences in cycle duration days, protocol type and gonadotropins dose, hCG day estradiol, serum progesterone level, and endometrial thickness between groups (Table II). Number of total and mature oocytes (MII), two pronuclei (2PN), total embryos, transferred embryos, quality of transferred embryos, and fertilization rate did not differ significantly between GCSF group and controls. There were no significant differences between groups in chemical, clinical and ongoing pregnancy rate, implantation rate, and miscarriage rate (Table III). 

**Table I:** Demographic characteristics of participants in two groups (n=50/each

**Characteristics**		**G-CSF Group**	**Control Group**	**p-value**
Age (Y) * #		31.24 ± 4.25	31.36 ± 5.15	0.89
Basal FSH level (day 3 FSH) (IU/L) * #		6.23 ± 2.20	6.36 ± 1.90	0.76
Previous embryo transfer (n) * #		0.36 ± 0.66	0.54 ± 0.88	0.25
Duration of infertility (Y) * #		6.5900 ± 4.09	7.29 ± 4.93	0.44
Type of infertility **$				1.00
	Primary	40 (80.0)	41 (82.0)	
	Secondary	10 (20.0)	9 (18.0)	
Etiology of infertility**$				0.80
	Male	29 (58.0)	31 (62.0)	
	Ovarian factor	10 (20.0)	8 (16.0)	
	Tubal	5 (10.0)	6 (12.0)	
	Unexplained	6 (12.0)	5 (10.0)	

FSH: Follicle-stimulating hormone

G-CSF: granulocyte colony-stimulating factor.

* Data are presented as mean±SD.

** Data are prersented as n(%).

# Student* t*-test

$ Chi-square test

**Table II:** Cycle characteristics of study patients in two groups (n=50/each

**Characteristics**		**G-CSF Group**	**Control Group**	**p-value**
hCG day estradiol (pg/ml) *#		1538.36 ± 1148.41	1757.57 ± 939.52	0.29
hCG day progesterone (pg/ml) *#		0.59 ± 0.48	0.66 ± 0.46	0.42
hCG day endometrial thickness(mm) *#		9.46 ± 1.71	9.62 ± 1.51	0.62
Duration of stimulation(days) *#		12.16 ± 1.69	12.28 ± 1.78	0.73
Gonadotropin dose (IU) *#		1675.75 ± 629	1819.74 ± 656	0.26
Protocol type**$				1.00
	Antagonist	49 (98.0)	49 (98.0)	
	Agonist	1 (2.0)	1 (2.0)	

G-CSF: granulocyte colony-stimulating factor

hCG=human chorionic gonadotropin

Note: Cycle characteristics were compared among the study group and the control group with the analysis of variance (ANOVA). If significant differences were found, each clinical diagnosis was compared with the control group to determine pairwise significance with Student’s t-test.

* Data are presented as mean±SD.

** Data are prersented as n(%).

# Student* t*-test

$ Chi-square test

**Table III:** IVF outcomes of study patients in two groups (n=50/each

**Characteristics**		**G-CSF Group**	**Control Group**	**p-value**
Oocytes Number *#		9.00 ± 4.25	10.00 ± 5.12	0.37
Mature Oocytes Number *#		7.00 ± 4.08	8.50 ± 4.73	0.64
2PN Number *#		4.00 ± 3.32	4.50 ± 3.66	0.66
Embryos Number *#		4.00 ± 2.98	4.00 ±3.64	0.84
Transferred Embryos Number *#		2.00 ± 0.70	2.00 ± .058	0.49
Fertilization rate*#		0.63 ± 0.25	0.66 ± 0.25	0.59
Implantation rate *#		0.12 ± 0.29	0.10 ± 0.24	0.76
Chemical pregnancy**$		9 (18.00)	10 (20.00)	1.00
Clinical pregnancy**$		9 (18.00)	9 (18.00)	1.00
Ongoing pregnancy**$		7 (14.00)	7 (14.00)	1.00
Miscarriage rate**$		2 (22.2)	3 (30.0)	0.71
Transferred Embryos quality**$				0.27
	A	19 (38.0)	17 (34.0)	
	B	28 (56.0)	25 (50.0)	
	C	3 (6.0)	8 (16.0)	

G-CSF: granulocyte colony-stimulating factor

2PN= Two pronuclei

* Data are presented as mean±SD.

** Data are prersented as n(%).

# Student* t*-test

$ Chi-square test

## Discussion

In the present study, G-CSF effect on implantation and pregnancy rates in normal infertile women candidate for IVF treatment were evaluated. It was found that pregnancy outcomes did not improve significantly after intra uterine G-CSF infusion in women with normal endometrial proliferation. G-CSF is a factor that rising the synchronization between uterine environment and embryo development during endometrial remodeling (15, 16). Previous studies have demonstrated that G-CSF can treat RIF and recurrent miscarriage by improving the inflammation process and endometrial receptivity (8-11).

In 2011, Gleicher *et al* represented a new option for thin endometrium treatment. They evaluated the G-CSF effect in four patients who underwent IVF that endometrial thickness had not increased with routine treatment. They reported successful endometrial thickness to at least 7 mm after G-CSF uterine infusion and all patients were conceived (12). Also, Tehraninejad *et al* in a study on fresh embryo transfer cycle in women with history of IVF cycle cancellation because of thin endometrium showed that the pregnancy chance and endometrial thickness was increased after G-CSF infusion (13).

While Eftekhar *et al* in their non-randomized clinical trial demonstrated that G-CSF improved implantation and clinical pregnancy rate in infertile women with thin endometrium in frozen-thawed embryo transfer cycles without improving endometrial thickness” (6). 

In the present study endometrial thickness in participants was in normal range (7-14 mm). We did not obtain significant differences between two groups in terms of chemical, clinical, ongoing pregnancy, implantation, and miscarriage rates. There are nor numerous studies on the effect of G-CSF in women with normal endometrial thickness. 

Similar to our results, Barad *et al* showed intrauterine G-CSF infusion in fresh embryo transfer cycles in IVF women with normal endometrial thickness do not affect endometrial thickness, implantation, and clinical pregnancy rates (7). Therefore it seems when there is evidence of impaired endometrial receptivity, like low thickness, RIF, or early miscarriage, G-CSF has beneficial effects on pregnancy and implantation rates. Transvaginal ultrasound assessment of endometrium can be used to determine preparation of the endometrium prior to embryo transfer. It is unclear that these assessments are helpful in determining whether the endometrium is optimally prepared (17).

A systematic review and meta-analysis of 14 studies shown that there may be a relationship between endometrial thickness and pregnancy, but implantation is more complex than be determined by single ultrasound (18). Now, during the treatment an infertile couple, the Endometrial Receptivity Array (ERA test) leads to the evaluation, at molecular level, of the endometrial factors (19). Therefore, it is suggested that for better G-CSF evaluation effects on endometrial receptivity and implantation, the molecular G-CSF effects e.g. integrins, proteomics, transcriptomics and ERA test be used in further studies (19, 20).

In summary, we showed that, in normal IVF women who had normal endometrium, the intrauterine infusion of G-CSF did not improve pregnancy outcomes. The available evidence does not support routine use of G-CSF in normal IVF women with normal endometrial thickness. More randomized controlled trials is needed for comparison of G-CSF effects on women with thin and normal endometrial thickness.

## Note

***Registration ID in IRCT:*** ***IRCT201508236420N13***
