# Correction to “Effect of granulocyte colony stimulating factor (G-CSF) on IVF outcomes in infertile women: An RCT” [Int J Reprod BioMed 2016; 14: 341-346]

**DOI:** 10.18502/ijrm.v20i1.10411

**Published:** 2022-02-18

**Authors:** Maryam Eftekhar, Robabe Hosseinisadat, Ramesh Baradaran, Elham Naghshineh

The authors of the article entitled “Effect of granulocyte colony stimulating factor (G-CSF) on IVF outcomes in infertile women: An RCT" have requested some corrections to their article due to further analysis of their data. The authors reviewed the data and confirmed that critical but inadvertent statistical analysis errors had occurred during the research. As the authors explained in their letter to the editor, the errors are listed as:

• The Mann-Whitney test has been added in the statistical analysis section.

• Some numbers in the tables have been changed and statistical tests have been revised as follows:

**Table 1:** Demographic characteristics of participants in two groups (n = 50/each)


**Characteristics**		**G-CSF group**	**Control group**	**p-value**
Age (Y) ${}^{*\#}$		31.24 ± 4.25	31.36 ± 5.15	0.89
Basal FSH level (day 3 FSH) (IU/L) ${}^{*\#}$		6.23 ± 2.20	6.36 ± 1.90	0.76
Previous embryo transfer (n) ${}^{*\#}$		0.36 ± 0.66	0.54 ± 0.88	0.25
Duration of infertility (Y) ${}^{*a}$		6.5900 ± 4.09	7.29 ± 4.93	0.66
Type of infertility ${}^{**}$ $			
	Primary	40 (80.0)	41 (82.0)	0.79
	Secondary	10 (20.0)	9 (18.0)	
Etiology of infertility ${}^{**\$}$			
	Male	29 (58.0)	31 (62.0)	
	Ovarian factor	10 (20.0)	8 (16.0)	
	Tubal	5 (10.0)	6 (12.0)	
	Unexplained	6 (12.0)	5 (10.0)	0.80
*Data are presented as Mean ± SD. **Data are prersented as n (%). # Student* t* test $ Chi-square test a: Mann-Whitney FSH: Follicle-stimulating hormone G-CSF: Granulocyte colony-stimulating factor				

**Table 2:** Cycle characteristics of study patients in two groups (n = 50/each)


**Characteristics**		**G-CSF group**	**Control group**	**p-value**
hCG day estradiol (pg/ml) ${}^{*a}$		1538.36 ± 1148.41	1757.57 ± 939.52	0.1
hCG day progesterone (pg/ml) ${}^{*a}$		0.59 ± 0.48	0.66 ± 0.46	0.25
hCG day endometrial thickness (mm) ${}^{*\#}$		9.46 ± 1.71	9.62 ± 1.51	0.62
Duration of stimulation (days) ${}^{*\#}$		12.16 ± 1.69	12.28 ± 1.78	0.73
Gonadotropin dose (IU) ${}^{*a}$		1675.75 ± 629	1819.74 ± 656	0.16
Protocol type ${}^{**b}$			
	Antagonist	49 (98.0)	49 (98.0)	
	Agonist	1 (2.0)	1 (2.0)	1.00
*Data are presented as Mean ± SD. **Data are prersented as n (%). # Student* t* test a: Mann-Whitney b: Fisher exact-test G-CSF: Granulocyte colony-stimulating factor hCG: Human chorionic gonadotropin Note: Cycle characteristics were compared among the study group and the control group with the analysis of variance (ANOVA). If significant differences were found, each clinical diagnosis was compared with the control group to determine pairwise significance with Student's *t* test				

**Table 3:** IVF outcomes of study patients in two groups (n = 50/each)


**Characteristics**		**G-CSF group**	**Control group**	**p-value**
Oocytes number ${}^{*\#}$		9.96 ± 4.25	10.98 ± 5.12	0.38
Mature oocytes number ${}^{*\#}$		8.26 ± 4.08	8.82 ± 4.73	0.64
2PN number ${}^{*\#}$		5.12 ± 3.329	5.58 ± 3.66	0.67
Embryos number ${}^{*\#}$		4.64 ± 2.98	5.14 ± 3.64	0.84
Transferred embryos number ${}^{*\#}$		2.14 ± 0.70	2.10 ± 0.058	0.5
Fertilization rate ${}^{*\#}$		0.63 ± 0.25	0.66 ± 0.25	0.52
Implantation rate ${}^{*\#}$		0.12 ± 0.29	0.10 ± 0.24	0.98
Chemical pregnancy ${}^{**\$}$		9 (18.00)	10 (20.00)	0.79
Clinical pregnancy ${}^{**\$}$		9 (18.00)	9 (18.00)	1.00
Ongoing pregnancy ${}^{**\$}$		7 (14.00)	7 (14.00)	1.00
Miscarriage rate ${}^{**\$}$		2 (22.2)	3 (30.0)	0.71
Transferred Embryos quality ${}^{**\$}$			0.27
	A	19 (38.0)	17 (34.0)	
	B	28 (56.0)	25 (50.0)	
	C	3 (6.0)	8 (16.0)	
*Data are presented as Mean ± SD. **Data are prersented as n (%). # Mann-whitny $ Chi-square test G-CSF: Granulocyte colony-stimulating factor 2PN= Two pronuclei				

One of the readers of the article informed the corresponding author that there seemed to be a number of errors in presenting the data, so the authors re-analyzed the data and due to the selection of an inappropriate statistical test, some numbers were incorrect. The corrected article has been provided with corrections to the paper and relevant tables. The authors have confirmed that there are no other errors. The corrected article has been reviewed by our editorial team, and we have confirmed that the overall conclusion has not been changed, as stated in the updated article available at: http://dx.doi.org/10.18502/ijrm.v14i5.747 (updated on 30 January 2022).

